# 500 Wound Coverage for Massive Burn Injuries ≥50% TBSA – A Systematic Review and Meta-Analysis

**DOI:** 10.1093/jbcr/irad045.097

**Published:** 2023-05-15

**Authors:** Gabriel Hundeshagen, Ulrich Kneser, Christian Tapking, Obada Abdulrazzak, Jack Palmer, Anamika Veeramani, Bianief Tchiloemba, Viola Stögner, Valentin Haug

**Affiliations:** BG Unfallklinik Ludwigshafen, University of Heidelberg, Ludwigshafen, Rheinland-Pfalz; BG Unfallklinik Ludwigshafen, University of Heidelberg, Ludwigshafen, Rheinland-Pfalz; BG Unfallklinik Ludwigshafen, University of Heidelberg, Ludwigshafen, Rheinland-Pfalz; The University of Arizona College of Medicine, Phoenix, Arizona; Boston University, Boston, Massachusetts; Harvard Medical School, Boston, Massachusetts; University of Calgary, Calgary, Alberta; Yale Plastic Surgery, New Haven, Connecticut; BG Unfallklinik Ludwigshafen, University of Heidelberg, Ludwigshafen, Rheinland-Pfalz; BG Unfallklinik Ludwigshafen, University of Heidelberg, Ludwigshafen, Rheinland-Pfalz; BG Unfallklinik Ludwigshafen, University of Heidelberg, Ludwigshafen, Rheinland-Pfalz; BG Unfallklinik Ludwigshafen, University of Heidelberg, Ludwigshafen, Rheinland-Pfalz; The University of Arizona College of Medicine, Phoenix, Arizona; Boston University, Boston, Massachusetts; Harvard Medical School, Boston, Massachusetts; University of Calgary, Calgary, Alberta; Yale Plastic Surgery, New Haven, Connecticut; BG Unfallklinik Ludwigshafen, University of Heidelberg, Ludwigshafen, Rheinland-Pfalz; BG Unfallklinik Ludwigshafen, University of Heidelberg, Ludwigshafen, Rheinland-Pfalz; BG Unfallklinik Ludwigshafen, University of Heidelberg, Ludwigshafen, Rheinland-Pfalz; BG Unfallklinik Ludwigshafen, University of Heidelberg, Ludwigshafen, Rheinland-Pfalz; The University of Arizona College of Medicine, Phoenix, Arizona; Boston University, Boston, Massachusetts; Harvard Medical School, Boston, Massachusetts; University of Calgary, Calgary, Alberta; Yale Plastic Surgery, New Haven, Connecticut; BG Unfallklinik Ludwigshafen, University of Heidelberg, Ludwigshafen, Rheinland-Pfalz; BG Unfallklinik Ludwigshafen, University of Heidelberg, Ludwigshafen, Rheinland-Pfalz; BG Unfallklinik Ludwigshafen, University of Heidelberg, Ludwigshafen, Rheinland-Pfalz; BG Unfallklinik Ludwigshafen, University of Heidelberg, Ludwigshafen, Rheinland-Pfalz; The University of Arizona College of Medicine, Phoenix, Arizona; Boston University, Boston, Massachusetts; Harvard Medical School, Boston, Massachusetts; University of Calgary, Calgary, Alberta; Yale Plastic Surgery, New Haven, Connecticut; BG Unfallklinik Ludwigshafen, University of Heidelberg, Ludwigshafen, Rheinland-Pfalz; BG Unfallklinik Ludwigshafen, University of Heidelberg, Ludwigshafen, Rheinland-Pfalz; BG Unfallklinik Ludwigshafen, University of Heidelberg, Ludwigshafen, Rheinland-Pfalz; BG Unfallklinik Ludwigshafen, University of Heidelberg, Ludwigshafen, Rheinland-Pfalz; The University of Arizona College of Medicine, Phoenix, Arizona; Boston University, Boston, Massachusetts; Harvard Medical School, Boston, Massachusetts; University of Calgary, Calgary, Alberta; Yale Plastic Surgery, New Haven, Connecticut; BG Unfallklinik Ludwigshafen, University of Heidelberg, Ludwigshafen, Rheinland-Pfalz; BG Unfallklinik Ludwigshafen, University of Heidelberg, Ludwigshafen, Rheinland-Pfalz; BG Unfallklinik Ludwigshafen, University of Heidelberg, Ludwigshafen, Rheinland-Pfalz; BG Unfallklinik Ludwigshafen, University of Heidelberg, Ludwigshafen, Rheinland-Pfalz; The University of Arizona College of Medicine, Phoenix, Arizona; Boston University, Boston, Massachusetts; Harvard Medical School, Boston, Massachusetts; University of Calgary, Calgary, Alberta; Yale Plastic Surgery, New Haven, Connecticut; BG Unfallklinik Ludwigshafen, University of Heidelberg, Ludwigshafen, Rheinland-Pfalz; BG Unfallklinik Ludwigshafen, University of Heidelberg, Ludwigshafen, Rheinland-Pfalz; BG Unfallklinik Ludwigshafen, University of Heidelberg, Ludwigshafen, Rheinland-Pfalz; BG Unfallklinik Ludwigshafen, University of Heidelberg, Ludwigshafen, Rheinland-Pfalz; The University of Arizona College of Medicine, Phoenix, Arizona; Boston University, Boston, Massachusetts; Harvard Medical School, Boston, Massachusetts; University of Calgary, Calgary, Alberta; Yale Plastic Surgery, New Haven, Connecticut; BG Unfallklinik Ludwigshafen, University of Heidelberg, Ludwigshafen, Rheinland-Pfalz; BG Unfallklinik Ludwigshafen, University of Heidelberg, Ludwigshafen, Rheinland-Pfalz; BG Unfallklinik Ludwigshafen, University of Heidelberg, Ludwigshafen, Rheinland-Pfalz; BG Unfallklinik Ludwigshafen, University of Heidelberg, Ludwigshafen, Rheinland-Pfalz; The University of Arizona College of Medicine, Phoenix, Arizona; Boston University, Boston, Massachusetts; Harvard Medical School, Boston, Massachusetts; University of Calgary, Calgary, Alberta; Yale Plastic Surgery, New Haven, Connecticut; BG Unfallklinik Ludwigshafen, University of Heidelberg, Ludwigshafen, Rheinland-Pfalz; BG Unfallklinik Ludwigshafen, University of Heidelberg, Ludwigshafen, Rheinland-Pfalz; BG Unfallklinik Ludwigshafen, University of Heidelberg, Ludwigshafen, Rheinland-Pfalz; BG Unfallklinik Ludwigshafen, University of Heidelberg, Ludwigshafen, Rheinland-Pfalz; The University of Arizona College of Medicine, Phoenix, Arizona; Boston University, Boston, Massachusetts; Harvard Medical School, Boston, Massachusetts; University of Calgary, Calgary, Alberta; Yale Plastic Surgery, New Haven, Connecticut; BG Unfallklinik Ludwigshafen, University of Heidelberg, Ludwigshafen, Rheinland-Pfalz

## Abstract

**Introduction:**

Early wound coverage remains one of the most essential variables influencing survival of extensively burned patients, especially with a total burned surface area (TBSA) of more than 50%. In patients with limited donor sites, techniques such as e.g. the Meek micrograft procedure or cultured epidermal allografts have proven to be viable methods. In this systematic review (SR) and meta-analysis (MA) we studied the outcomes of different techniques for wound coverage in patients with massive burn injuries ≥50% TBSA within the last 17 years.

**Methods:**

According to PRISMA guideline the medical databases PubMed, Cochrane, Embase, Web of Science Medical records were screened in 5 languages from 01/2005 until 01/2022 by three reviewers, independently. Inclusion criteria were prospective or retrospective studies on patients with massive burns of ≥50% TBSA.

**Results:**

After a two-stage review process, 33 studies were identified for the SR and MA. Regions of publication were Asia (n=15), North America (n=8), Europe (n=7), Australia (n=3). In total, 1678 patients with a mean age of 31.4 ± 14.2 years were included. The male to female ratio was 2.25 : 1. Mean TBSA was 66,5 ± 12.2%. Methods of wound coverage consisted of Meek micrografts, cultured epithelial autografts and/or allografts. The mean length of stay was 76.5 ± 32.4 days. Initial graft-take was 78,0 ± 15.8%. Grafting techniques did neither substantially differ in the length of stay nor the healing rate.

**Conclusions:**

In patients with massive burn injuries of ≥50% TBSA the length of stay and the healing rates were comparable between Meek micrograft procedure, cultured epithelial allografts and combined techniques.

**Applicability of Research to Practice:**

Wound coverage by Meek micrograft procedure with expansion of up to 1:10 and a potential combination with cultured epithelial allografts should be considered early in patients with massive burn injury.

